# The gut at war: the consequences of enteropathogenic *Escherichia coli* infection as a factor of diarrhea and malnutrition

**DOI:** 10.1590/S1516-31802000000100006

**Published:** 2000-01-02

**Authors:** Ulysses Fagundes, Isabel Cristina Affonso Scaletsky

**Keywords:** *Escherichia coli*, Infantile diarrhea, Lethality risk, Persistent Diarrhea, Acute Diarrhea, Escherichia Coli, Diarréia infantil, Risco de mortalidade, Diarréia persistente, Diarréia aguda

## Abstract

Diarrheal disease is still the most prevalent and important public health problem in developing countries, despite advances in knowledge, understanding, and management that have occurred over recent years. Diarrhea is the leading cause of death in children under 5 years of age. The impact of diarrheal diseases is more severe in the earliest periods of life, when taking into account both the numbers of episodes per year and hospital admission rates. This narrative review focuses on one of the major driving forces that attack the host, namely the enteropathogenic *Escherichia coli* (EPEC) and the consequences that generate malnutrition in an early phase of life. EPEC serotypes form dense microcolonies on the surface of tissue-culture cells in a pattern known as localized adherence (LA). When EPEC strains adhere to epithelial cells in *vitro or in vivo* they cause characteristic changes known as Attaching and Effacement (A/E) lesions. Surface abnormalities of the small intestinal mucosa shown by scanning electron microscopy in infants with persistent diarrhea, although non-specific, are intense enough to justify the severity of the clinical aspects displayed in a very young phase in life. Decrease in number and height of microvilli, blunting of borders of enterocytes, loss of the glycocalyx, shortening of villi and presence of a mucus pseudomembrane coating the mucosal surface were the abnormalities observed in the majority of patients. These ultrastructural derangements may be due to an association of the enteric enteropathogenic agent that triggers the diarrheic process and the onset of food intolerance responsible for perpetuation of diarrhea. An aggressive therapeutic approach based on appropriate nutritional support, especially the utilization of human milk and/or lactose-free protein hydrolyzate-based formulas and the adequate correction of the fecal losses, is required to allow complete recovery from the damage caused by this devastating enteropathogenic agent.

## resumo

**Resumo:**

A doença diarréica ainda é o problema mais prevalente é importante na saúde pública dos países em desenvolvimento, a despeito dos avanços nos conhecimentos da fisiopatologia e do manejo clínico que vem ocorrendo ao longo destes últimos anos. Diarréia é a principal causo de morte entre as crianças menores de 5 anos nas populações de baixa renda dos países em desenvolvimento. O impacto da doença diarréica faz-se notar de forma mais intensa nos períodos mais precoces da vida, em especial no primeiro ano, quando são levados em consideração o número de episódios de diarréia por ano e as taxas de admissão hospitalar. Esta revisão tem o escopo de enfatizar um dentre os inúmeros agentes enteropatogênicos que agridem o hospedeiro, particularmente os sorogrupos enteropatogênicos clássicos da Escherichia coli (EPEC) e suas respectivas conseqüências que levam ao agravo nutricional em fases precoces da vida. Os sorogrupos de EPEC formam densas microcolônias sobre a superfície de células epiteliais mantidas em cultura de tecido determinando um padrão de comportamento denominado aderência localizada (AL). Quando as cepas de EPEC aderem a células epiteliais *in vivo ou in vitro* provocam alterações características conhecidas como "Attaching" e "Effacement’ (A/E). As alterações da superfície da mucosa do intestino delgado visualizadas pelo microscópio eletrônico de varredura em lactentes com diarréia persistente, embora não específicas, são suficientemente intensas para justificar a gravidade dos aspectos clínicos observados. Diminuição no número e na altura das microvilosidades, borramento dos limites dos enterócitos, perda do glicocálice, diminuição do número de vilosidades e a presença de uma pseudomembrana recobrindo a superfície mucosa, foram as anormalidades descritas na maioria dos pacientes. Essas altarações ultraestruturais podem ser devidas à associação entre o agente Woccioso que deflagra o processo diarréico e a instalação de intolerâncias alimentares que perpetuam a diarréia. Uma abordagem terapêutica agressiva, baseada em suporte nutricional adequado, em especial utilizando-se leite humano e/ou fórmulas a base de hidrolisado protéico, além da correção das perdas hidroeletrolíticas fecais, é necessária para proporcionar a recuperação clínica e nutricional nessa enfermidade.

Malnutrition and diarrhea are manifestations of a real war in the gut. They can be considered to be an even more dramatic hazard because the enemy is invisible (enteropathogenic microorganisms) and this hazard is widespread everywhere in communities where there are high levels of environmental contamination. Although this is not a conventionally known field of battle, the consequences are more devastating. The combat occurs within the organism of the host, that is, the intestinal lumen. It never stops and it only ends when the host evolves to death. This review focuses on one of the major driving forces that attack the host, namely enteropathogenic *Escherichia coli* (EPEC), and the consequences that generate malnutrition in an early phase of life.

Diarrheal disease is still the most prevalent and important public health problem in developing countries, despite advances in knowledge, understanding, and management that have occurred over recent years. Diarrhea is the leading cause of death in children under 5 years of age.^1^ The impact of diarrheal diseases is more severe in the earliest periods of life, when taking into account both the number of episodes per year and hospital admission rates. The first estimate of worldwide morbidity and mortality from diarrheal disease was based on active surveillance data collected from 24 selected longitudinal studies of children undertaken over 3 decades, and was published in the early 1980s.^2^ It was a landmark report which showed that, based on 1980 population estimates, there were 744 million to 1 billion episodes of diarrhea and 4.6 million deaths each year from diarrheal disease in children under 5 years of age in Africa, Asia and Latin America. A decade later, improved case management had helped improve survival rates such that global mortality was lower (3.3 million deaths per year; estimate range = 1.5 to 5.1 million), but the incidence of diarrhea (2.6 episodes per child per year) had remained virtually unchanged.^3^

Current knowledge concerning fluid and electrolyte losses and their replacement during an acute episode of diarrhea has proved to be an excellent tool for the treatment of the first stage of the disease, especially at community level. However, in those more severe cases that require hospitalization, the risk of perpetuation of diarrhea is beyond the efficacy of oral hydration, since food intolerance and nutritional aggravation are the most frequent clinical complications.^4^ In order to propose guidelines aiming towards a significant decrease in infantile mortality rates, a better understanding of the interaction between the enteropathogenic agents and the host would seem to be highly desirable.

Taking into account the importance of the problem, especially in hospitalized patients, a project was designed to determine the epidemiological and clinical factors potentially associated with lethality in infants due to severe acute diarrhea.^5^ Five hundred and eleven children under 5 years of age, hospitalized due to severe diarrhea in our Metabolic Unit, were prospectively studied. All patients had been suffering from diarrhea lasting for less than 14 days at the time of admission. The patients’ mean age was 5.5 months; 87.5% were under 1 year of age and 75.0% were under 6 months of age. Patients were divided into two groups according to the clinical evolution during hospitalization:

**Table:** 

Group I -	17 (3.3%) infants who died; mean age was 3.8 months.
Group II -	494 (96.7%) infants who had a satisfactory evolution and were discharged from hospital in good clinical conditions; mean age was 7.1 months.

The following parameters were evaluated in relation to the patients’ clinical outcome: birth weight, sex, age, duration of diarrhea prior to hospital admission, nutritional status, hydration conditions, enteropathogenic agents identified in the stool cultures, food intolerance and duration of hospitalization. The following variables showed a significant association with risk factor for death (RFD): age, presence of EPEC in the stool culture, severe protein-calorie malnutrition (PCM III) and food intolerance (Table 1).

**Table 1:** Variables that showed a significant association with death in infants hospitalized due to severe acute diarrhea

Evaluated Variable	Group I^1^ *(n = 17)*	Group II ^2^ *(n = 494)*	% Total^3^	EV/Death *(%)^4^*	Death/EV *(%)^5^*	RR^6^
Age<6 months	15	320	2.9	88.2	4.5	3.9
EPEC in the stools	9	118	1.8	52.9	7.1	3.4
PCM III	7	62	1.4	41.2	10.1	3.6
Food Intolerance	11	194	2.1	64.7	5.4	2.7

EV = Evaluated variable; 1 - Death; 2 - Survival; 3 - Percentage of patients that presented the evaluated factor and died; 4 - Percentage of patients that presented the evaluated factor and died in relation to the total of deaths; 5 - Percentage of deaths (patients that presented the evaluated factor); 6 - Relative risk

RFD was 3.9 times higher for infants under 6 months of age (4.5% vs. 1.1%) (P < 0.05). EPEC was isolated in the stool culture in 9 (52.9%) patients belonging to Group I and in 118 (23.9%) belonging to Group II. Evaluation of the nutritional status revealed that only 143 (28%) patients were well nourished when admitted to the hospital. The remaining 368 (72%) mal-nourished patients were classified as follows: protein-calorie malnutrition (PCM) I 174 (34%), PCM II 125 (24.5%) and PCM III 69 (13.5%). RFD was 3.6 times higher for patients suffering from severe malnutrition in comparison with well-nourished ones and those suffering moderate degrees of malnutrition, at the time of hospital admission (P < 0.05). RFD was 3.4 times higher in those cases in which some EPEC serogroups were identified in the stools in comparison with patients whose etiologic investigation was negative. Food intolerance was detected in 11 (64.7%) patients belonging to Group I and in 194 (39.7%) belonging to Group II. RFD was 2.7 times higher for patients who revealed food intolerance in comparison to those who did not present this clinical complication. Frequency of food intolerance was higher and statistically significant, in patients under 6 months in comparison to those above 6 months of age, respectively: 53.9% vs. 27.1% (P < 0.01).

In the present study, stool culture was performed in 461 patients and an enteropathogenic agent was isolated in 253 (54.9%) of them. Distribution of the different enteropathogenic agents according to the clinical outcome of patients is shown in Table 2. EPEC serogroups were the most frequent agents identified in the stools (27.5%), especially the following specific serogroups: O111 (19.3%) and O119 (5.2%). In Group I, the etiologic investigation was positive in 75% of the cases, and EPEC serogroups were the most frequent, present in 56.3% of the cases, followed by *Shigella* (12.5%). In Group II, the etiologic investigation was positive in 54.1% of the cases, and EPEC serogroups were also the most frequent enteropathogenic agents, in 42.2% of the cases, and again followed by *Shigella* (7.6%).

**Table 2:** Distribution of the different enteropathogenic agents according to the clinical outcome of the patients

Enteropathogenic agents	Death	Survival	Total
EPEC	9 (7.1)	118	127
Shigella	2 (5.9)	34	36
Salmonella	0 (0)	16	16
EIEC	0 (0)	3	3
ETEC	0 (0)	5	5
Campylobacter	0 (0)	20	20
Cryptosporidium	0 (0)	2	2
Rotavirus	0 (0)	13	13
Mixed infection	1 (3.2)	30	31
Negative	4(2.4)	204	208
**Total**	**17**	**494**	**511**

EPEC = Enteropathogenic Escherichia coli; ETEC = Enterotoxigenic *Escherichia coli;* EHEC = Enterohemorragic *Escherichia coli;* EIEC = Enteroinvasive *Escherichia coli;* GROUP I – Death; GROUP II – Survival

The associative comparison between age groups, under or over 6 months, and the presence of EPEC serogroups in the stool culture was positive in 33.2% of patients under 6 months of age and in 21.7% of patients over 6 months of age, respectively. There was also a significant positive association between identification of EPEC serogroups in the stool culture and presence of food intolerance. Considering the usual scanty resources available in developing countries, a reduction in diarrhea-related mortality may be feasible by identifying high-risk subjects and targeting them for intensive intervention. In the present study we were able to identify four factors significantly associated with death, namely: age, severe malnutrition (PCM III), presence of EPEC in stool cultures and food intolerance.

EPEC infections are unusual in most industrialized countries, but they continue to be important enteric pathogens in developing countries, especially in large urban centers, affecting non-breast-fed infants belonging to low-income families that live in unfavorable conditions, lacking water supply and sewage systems.

In São Paulo, Brazil, EPEC strains are the major enteropathogenic agents in infants under 1 year of age, the highest prevalence occurring in those under 6 months of age.^6^ This pattern of prevalence has been practically unchanged for at least the last 40 years. Since the very first reports of investigation of the etiology of gastroenteritis performed in São Paulo in the late 1950s, EPEC strains have emerged as the most important bacterial enteropathogenic agent in infants belonging to families in low socioeconomic levels.^7^

São Paulo, the largest city in South America with its 15 million inhabitants, represents a very typical example of this prominent problem of public health, frequently observed in overcrowded urban centers of developing countries. São Paulo is the greatest industrial and financial center in Brazil, as well as in the whole of Latin America. However, an unbalanced distribution of wealth engenders the coexistence of an opulent upper middle class, which indulges in cultural, social and sporting activities all year round, with an extremely impoverished population struggling to survive. Unfortunately, the latter represents a significant proportion of the inhabitants of the city and most of them, because of their lack of economic resources, are compelled to live in our slums (*favelas*). On account of their very low income and absence of professional skills, these people live under wretched conditions in the peripheral areas of the city. Houses in the slums are made of wood or clay, there is no sewage system, and excrement collects in the streams generally present along the edges of the slums. The lack of appropriate sanitary conditions favors the existence of high levels of environmental contamination affecting children, especially infants, and leads to a high incidence of enteric infections. EPEC strains are prone to becoming very conspicuous in communities where there are elevated degrees of promiscuity.

This classic pattern of etiological distribution of enteropathogenic agents is seen in various other urban centers in developing countries, for example, in Brasilia. This is the present capital of the country and it was designed, in the early 1950s, to accommodate a population no greater than 600,000 inhabitants, but its growth got out of control and the population has now reached 2,000,000 people. This astounding increase in the population size has led to a wealth distribution pattern very similar to that experienced in São Paulo, with the creation of numerous slums on the outskirts of the city.

Considering these biosocial characteristics, a study was undertaken in Brasilia to investigate the epidemiological and clinical features of acute diarrhea caused by EPEC strains in comparison to those caused by other enteric pathogens.^8^ During a two-year period, 200 infants under 2 years of age with acute diarrhea lasting less than 6 days were compared to 40 healthy infants matched for age. Patients were followed for 4 weeks after discharge from hospital. Stool samples were obtained for detection of bacterial, viral and protozoan enteric agents.

EPEC was isolated in stool cultures of 84 (42.0%) case children. From the 40 control children EPEC was isolated from 9 (22.5%). The etiologic agents of acute diarrhea among other case children are shown in Table 3. After EPEC, the most frequent cause of acute diarrhea was rotavirus, which was not detected in any of the control children. EPEC strains were identified in the stools of the 29 out of 32 children with mixed infection. Agents most frequently isolated along with EPEC were: rotavirus, *Campylobacter, Shigella sonnei, Shigella flexneri, Giardia lamblia* and *Entamoeba histolytica*. Table 4 shows the occurrence of the several EPEC serogroups identified in stools of children belonging to both groups. Serogroups O111, O142, and O119 were statistically more often isolated from cases than from control children.

**Table 3:** Enteropathogenic agents isolated in the stools of 200 infants with acute diarrhea (cases) and in 40 age-matched children without diarrhea (controls)

Etiological Agent	Cases n (%)	Controls n (%)
Enteropathogenic *E. coli*	84 (42.0)	9 (22.5)
*Shigella* spp	24 (12.0)	0
*Campylobacter* spp	22 (11.0)	0
*Salmonella* spp	8 (4.0)	0
Enteroinvasive *E. coli*	2 (1.0)	0
Rotavirus	32 (16.0)	0
*Entamoeba histolytica*	26 (16.0)	1 (2.5)
*Giardia lamblia*	12 (6.0)	2 (5.0)
*Cryptosporidium*	1 (0.5)	0
Mixed Infections	32 (16.0)	1 (2.5)

**Table 4:** Enteropathogenic *E. coli* serogroups identified in the stool of 200 children with acute diarrhea (cases) and 40 children without acute diarrhea (controls)

EPEC Serogroups	Cases n (%)	Controls n (%)	P-Value
O111	25 (12.5)	0	< 0.001
O142	18 (9.0)	2 (5.0)	< 0.05
O119	10 (5.0)	0	< 0.01
O55	5 (2.5)	0	NS
O158	5 (2.5)	1 (2.5)	NS
O125	5 (2.5)	1 (2.5)	NS
O26	5 (2.5)	1 (2.5)	NS
O127	4 (2.0)	1 (2.5)	NS
O126	3 (1.5)	3 (7.5)	NS
O128	2 (1.0)	0	NS
O18	1 (0.5)	0	NS
O114	1 (0.5)	0	NS

NS = not significant

Table 5 shows a comparison between the clinical characteristics of case children with EPEC as the sole agent and case children with other etiologic agents. The mean age of children with EPEC was 8.3 months, 78.2% of whom were under 12 months of age and 45.2% of whom were under 6 months of age. Oral rehydration was utilized in 80 (95.2%) of EPEC patients, which resulted in correction of dehydration in 59 (73.7%), whereas in the other diarrhea cases, oral rehydration was successful in 104 (89.7%) patients (P < 0.01). The main causes of oral rehydration failure among the remaining 21 (26.3%) EPEC patients were: persistent vomiting,^3^ metabolic acidosis and severe dehydration,^2^ hypokalemia,^3^ seizure,^2^ and recurrent episodes of dehydration.^11^ Food intolerance was the major gastrointestinal complication and also the most important cause of persistence of diarrhea. Cow's milk intolerance occurred in 23 of 74 (31.1%) patients of the EPEC group and in 10 (9.8%) in the other pathogens group (P < 0.001). The mean duration of diarrhea among infants with EPEC was 11.7 days while in the other group it was 7.1 days (P < 0.05). Diarrhea lasted more than 14 days in 21 of 71 (28.4%) patients in the EPEC group whereas in the other group diarrhea lasted more than 2 weeks in 7 (6.9%) patients (P < 0.01).

**Table 5:** Comparison of clinical features of acute diarrhea caused by enteropathogenic *E. coli* and other pathogens

Variables	EPEC (n = 84)	Others (n = 116)	P-Value	Odds Ratio (95% CI)
Mean age (months)	8.3	8.4	NS	
Mean duration of diarrhea (days)	3.4	3.1	NS	
Dehydration				
Grade I	65 (77.4%)	102 (87.9%)	NS	
Grade II	15 (17.8%)	13 (11.2%)	NS	
Grade III	4 (4.8%)	1 (0.9%)	NS	
ORT Failure	21* (26.3%)	12 (10.3%)	< 0.01	3.08 (1.33 to 7.22)
Hospitalization	26+ (34.2%)	13 (11.2%)	< 0.01	4.12 (1.84 to 9.32)
Mean duration of hospitalization	5.9	5.8	NS	
Cow's milk intolerance	23‡ (31.1%)	10§ (9.8%)	< 0.001	4.15 (1.72 to 10.21)
Mean duration of disease (days)	11.7	7.1	< 0.05	
Persistent diarrhea	21‡ (28.4%)	7§ (6.9%)	< 0.01	5.38 (2.00 to 15.00)

ORT = oral rehydration therapy;

* Number of patients in the EPEC group that received ORT = 80; + Number of patients in the EPEC group available for calculation = 76;

‡ Number of patients in the EPEC group who completed the follow-up evaluation = 74;

§ Number of patients in the other diarrhea group who completed the follow-up evaluation = 102

In summary, EPEC strains can induce copious secretory diarrhea with intense fluid and electrolyte losses in stools that may require intravenous rehydration. Moreover, EPEC strains can also provoke severe malabsorption of nutrients, including the glucose component of oral rehydration solutions, and in a second stage of the disease this may even evolve to food intolerance that results in nutritional aggravation and persistence of diarrhea.

EPEC serotypes form dense microcolonies on the surface of tissue-culture cells in a pattern known as localized adherence (LA).^9^ This behavior is encoded by a large plasmid that is common to specific sero-types of these strains and is associated with the production of type IV fimbriae known as bundle-forming-pili (BFP).^10^ When EPEC strains adhere to epithelial cells *in vitro* or *in vivo* they cause characteristic changes known as Attaching and Effacement (A/E) lesions. Effacement refers to the loss of host cell microvilli. The epithelial membrane beneath the adherent organisms is raised locally to form pedestals. The cytoskeletons of the host cells are responsible for the formation of pedestals and probably also for the effacement of the microvilli. Several cytoskeletal components accumulate in the epithelial cells beneath the adherent EPEC. The role of microvilli effacement and pedestal formation in disease remains undefined. The determinants of A/E lesion formation have been localized to a large island of pathogenicity on EPEC chromosomes termed the locus of enterocyte effacement or LEE.^11^

We were indeed able to confirm this hypothesis in vivo by studying the nutritional impact of small bowel ultrastructural alterations in 3 infants with severe gastroenteritis caused by 3 different EPEC sero-types, namely O111, O119 and O18.^12^ The infants were hospitalized due to profuse watery diarrhea lasting less than 6 days (Table 6).

**Table 6:** Clinical features of the patients with diarrhea caused by serogroups of enteropathogenic *Escherichia coli*

Patient	Age (months)	Weight* (g)	Days of diarrhea§	No. and type of stools/day	IV hydration (days)	Duration of diarrhea(days)	Stool culture
1	3	5300	5	5 – watery	5	15	EPEC O111
2	3	5200	5	10 – watery	2	15	EPEC O119
3	1	5500	4	6 – watery	2	12	EPEC O18

* Weight after fluid replacement;

§ Before hospitalization

After dehydration was reversed, patients were fed full strength cow's milk formula *ad libitum*. During the first 3 days of formula feedings diarrhea persisted and was associated with weight loss. Stools contained reducing substances and fecal pH was below 6.0. A casein based, lactose-free formula was therefore introduced. As there was persistence of symptoms, a protein hydrolyzate, lactose-free formula was fed on the sixth day of hospitalization. After this dietary modification, diarrhea ceased and patients started gaining weight. The z-score values obtained on admission, when initiating the protein-hydrolyzate formula, and on day 30 of the follow-up are shown in Table 7. At the end of day 30 of the follow-up, the patients showed an average daily weight gain of 26.4 grams from the time that feeding with the protein-hydrolyzate formula was started.

**Table 7:** Evolution of the nutritional status of the patients determined by the z-score system (weight-for-age)

Patient	Z - SCORE *		
	Time 0	Time 1	Time 2
1	¾ 0.72	¾ 1.68	¾ 1.27
2	¾ 0.82	¾ 1.40	¾ 1.35
3	¾ 1.19	¾ 1.75	¾ 1.72

* Time 0 – 1^st^ day of hospitalization; Time 1 – Introduction of proteinhydrolyzate, lactose-free formula (6^th^ day of hospitalization); Time 2 – After 30 days of follow-up

On the sixth day of hospitalization, due to persistence of diarrhea, a small bowel biopsy was performed. Optical microscopy analysis revealed an intense villous atrophy and the presence of numerous Gram-negative bacteria adhered to the apical portion of the enterocytes. Electron microscopy study showed severe ultrastructural derangements of the small bowel epithelium. Bacteria could be seen attached to long disordered microvilli, occasionally settled on the apical surface on pedestals and even within cytoplasmic phagocytic vacuoles. Effacement of microvilli and pedestal formation at sites of attachment were also observed. Bacteria were also seen in the interior of the enterocyte as well as several multivesicular bodies. Ultrastructural lesions are usually associated with the presence of clusters of bacteria adhering to the microvilli, but such an association is not always found, and some focal dissolution of the microvilli can be seen even in the absence of bacteria. These ultrastructural derangements have been described along with other serogroups, namely O125 and O114, in infants with chronic diarrhea and malnutrition. In the current study we were able to demonstrate that the ultrastructural lesions may already occur in an early phase of infection, triggering persistence of diarrhea and nutritional aggravation.

EPEC is one of the most frequent enteropathogenic agents identified in stools of infants with persistent diarrhea.^13^ Persistent diarrhea, of presumably infectious etiology, characterized by lasting for more than 14 days, is responsible for approximately half of the 3,300,000 yearly deaths due to diarrhea in developing countries.^14^ It is accepted that 3 to 23% of acute diarrhea episodes evolve to persistent diarrhea^15^ and the other enteropathogenic agents involved with persistent diarrhea are: *Salmonella*, *Campylobacter* and *Rotavirus*.^16^ Recently, some new other agents such as *Cryptosporidium*, enteroaggregative *Escherichia* *coli* (EAggEC) and *Giardia lamblia* have been reported to be associated with persistent diarrhea.^17^

Optical microscopy studies of the small bowel mucosa in infants with persistent diarrhea have shown different degrees of villous atrophy, including subtotal villous atrophy with increased inflammatory infiltrate in the lamina propria, frequently the pattern of alteration of intestinal morphology.^18^ These small bowel lesions in persistent diarrhea may be due to several noxious factors, namely nutritional deficiencies, direct action of some enteropathogenic agents on the enterocyte and food allergy, which can in isolation or in association contribute to perpetuation of diarrhea.

Furthermore, ultrastructural derangements of enterocytes and organelles, observed by utilizing transmission electron microscopy, have been reported in infants suffering from persistent diarrhea.^19^ We have already shown that there are a great variety of alterations in the enterocytes. Tuft formation among microvilli in focal areas and an increased number of multivesicular bodies were very frequently observed. Microvilli could also be seen to be reduced in number and height and occasionally the cell surfaces were seen to be completely denuded. Mitochondria appeared to lose inner matrix density and in some specimens they were swollen and the endoplasmic reticulum was vacuolated.

Considering the importance of alterations of the small bowel epithelium in the genesis of perpetuation of diarrhea and malnutrition, a study was designed in order to investigate the ultrastructural features of the small bowel surface in malnourished infants with persistent diarrhea utilizing scanning electron microscopy.^20^

Sixteen infants with persistent diarrhea consecutively admitted to our Metabolic Unit were studied. The age range of patients was 2 to 10 months, mean 4.8 months. All patients had diarrhea lasting for more than 14 and less than 30 days. The major clinical characteristics of the patients with persistent diarrhea are shown in Table 8.

**Table 8:** Persistent diarrhea: bacterial culture of the jejunal secretion and stools

Patient	Jejunal secretion	Stool
1	*Escherichia coli*	*
2	*Escherichia coli* + *Pseudomonas aeruginosa*	*
3	Sterile	*
4	EPEC O119: H6	EPEC O119:H6+EAggEC
5	Sterile	*
6	*Escherichia coli* + *Pseudomanas aeruginosa* + *Klebisiella pneumoniae*	EPEC O111:H
7	Sterile	*
8	*Pseudomonas Aeruginosa*	EAggEC
9	*Enterobacter* spp	*Shigella flexneri*
10	*Pseudomonas aeruginosa* + *Enterobacter* spp	*
11	*Escherichia coli*	EPEC O111:H2+EAggEC
12	Sterile	*
13	*Escherichia coli*	EPEC O111:H2
14	EPEC O55	*Shigella Sonnei* + EPEC 055 + EPEC O111:H2 + EAggEC
15	EPEC 0119:H6	EPEC 0119:H6
16	Sterile	*

* Failure to isolate an enteropathogenic agent

Enteropathogenic agents were identified in the stools of 8 (50.0%) patients and bacterial proliferation in the intestinal lumen was present in 67.8% of the patients, most of which were considered to be of the colonic type of microflora (Table 9). Bacterial over-growth in the small bowel lumen may induce deconjugation and 7-alpha dehydroxylation of the primary bile salts, leading to morphological damage of the jejunal mucosa, secretion of sodium and water, malabsorption of nutrients, and rupture of the intestinal permeability barrier.^21,22^ The latter favors the penetration of intact macromolecules, thus potentially leading to food allergy.^23^

**Figure 1:**
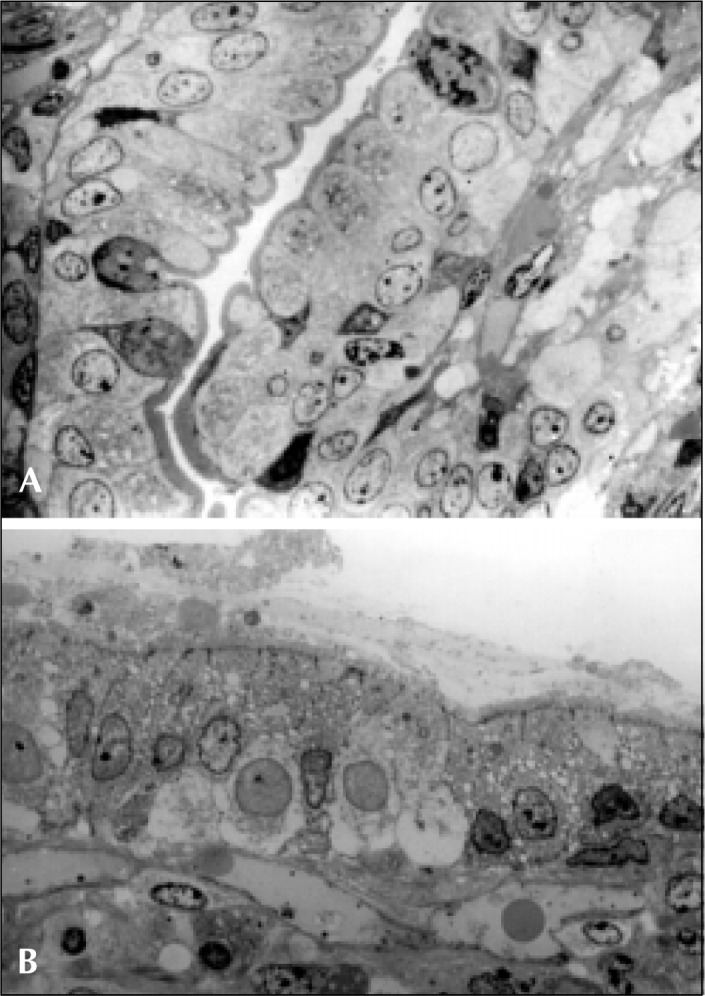
A) Optical microscopy of semi-thin section of the duodenum of a control. The enterocytes are well differentiated, with regular nuclei. The microvilli are totally preserved in shape and number; B) Optical microscopy of a semi-thin section of the duodenum of a patient with persistent diarrhea. The epithelium shows shortened microvilli, increased infiltrate of lymphocytes and eosinophils, intracytoplasmatic vacuolization and the presence of multivesicular bodies. Increased infiltrate of eosinophils in the lamina propria is also seen.

Morphological abnormalities in the small bowel mucosa were observed in all patients, varying in intensity from moderate to severe, when semi-thin sections were analyzed under the optical microscope. Moderate villous atrophy was the most frequent pattern, observed in 56.2% of the jejunal specimens studied, while sub-total villous atrophy was observed in the remaining patients. Patchy areas of blunted microvilli were frequently seen associated with intracytoplasmatic vacuolization. The inflammatory infiltrate in the lamina propria was increased in intensity denoting the presence of lymphocytes, plasma cells and eosinophils. In contrast, controls showed finger-like villi, microvilli were totally preserved in shape and number and intracytoplasmatic organelles had a completely normal appearance (Figure 1).

**Figure 2:**
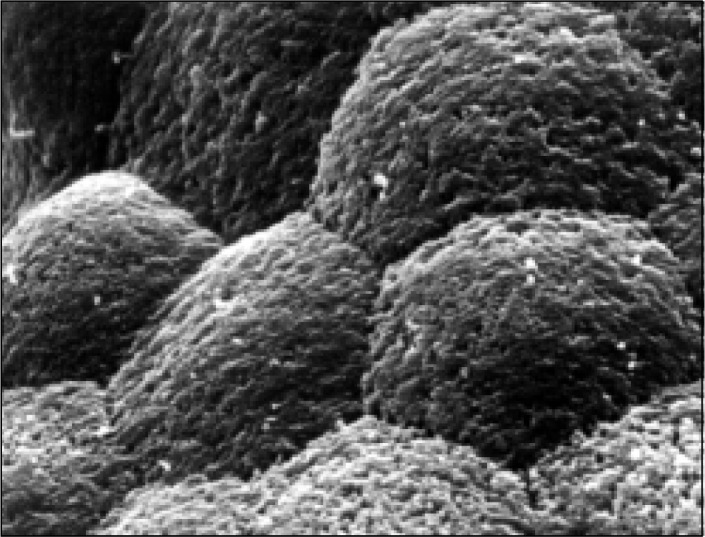
Scanning electron microscopy of the duodenum of a control. Enterocytes are well preserved, clearly delimited, with individual borders well visible. The cells show a flower-like arrangement.

Under the scanning electron microscope at low power, control biopsies showed leaf and finger-shaped villi. Mucus strands were predominant at the periphery of the biopsies, mucus being present to a lesser degree between the villi. At higher power the enterocytes were clearly visible, cell borders were sharply outlined, microvilli were well preserved and cells showed a flower-like arrangement (Figure 2).

**Figure 3:**
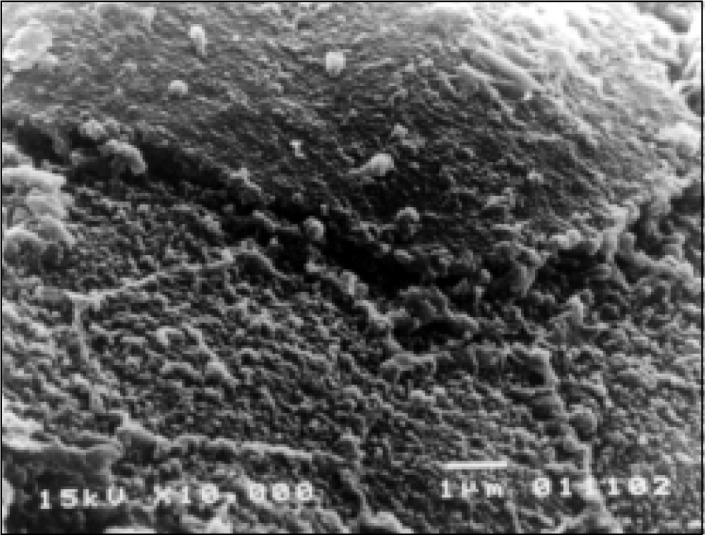
Scanning electron microscopy of the duodenum of a patient with persistent diarrhea. Enterocytes are distorted in appearance and microvilli are shortened in height. Tips of the microvilli are well visible because the absence of the glycocalyx.

**Figure 4:**
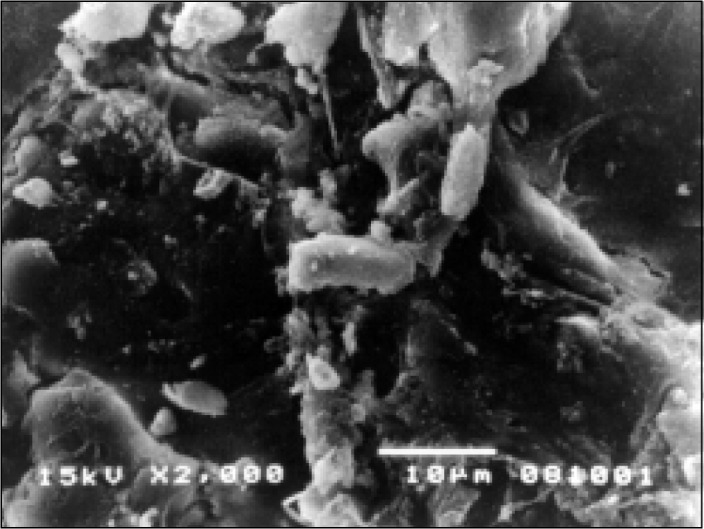
Scanning electron microscopy of the duodenum of a patient with persistent diarrhea. Cell borders are not well visible and a bacilliform microorganism is seen tightly adhering to the epithelial surface surrounded by particles of mucus.

On the other hand, all patients with persistent diarrhea showed numerous abnormalities of the small bowel surface, although these varied in intensity. At lower power, most of the villi showed mild to moderate stunting, but on several occasions there was subtotal villous atrophy. The high-power photomicro-graphs confirmed derangement of the enterocytes, frequently cell borders were not clearly defined, very often microvilli were decreased in number and height and in some areas there was total disappearance of the microvilli (Figure 3). Microorganisms with a bacilliform configuration, tightly adhering to the enterocytes, were seen in some cases, as well as the presence of lymphocytes and fat droplets overlying the surfaces of the enterocytes (Figure 4).

**Figure 5:**
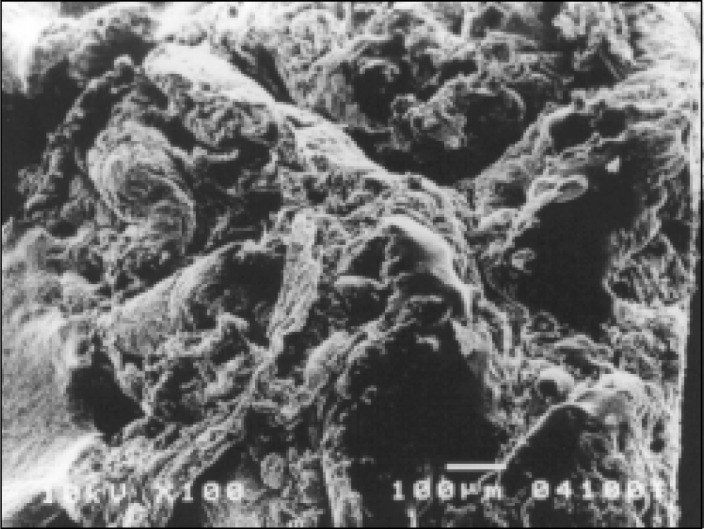
Scanning electron microscopy of the duodenum of a patient with persistent diarrhea. Microvilli surfaces are partly covered with a mucus-fibrinoid pseudomembrane.

In half of the patients a mucous-fibrinoid pseudomembrane partially coating the enterocytes was observed (Figure 5). The mucus coating may hamper absorption of the nutrients of the diet due to mechanical block, thus leading to osmotic diarrhea and nutritional aggravation. This hypothesis may be supported by the finding of fat droplets accumulated on the apical surfaces of the enterocytes. Taking these considerations as being pertinent, fat malabsorption could be explained by at least two different mechanisms: (1) a decrease in the bile salt pool as a consequence of bacterial proliferation resulting in deconjugation and 7μ-dehydroxylation of the primary bile salts;^21^ (2) the presence of the mucus-fibrinoid pseudomembrane acting as a mechanical block avoiding the passage of dietary fat to the interior of the enterocytes.

Surface abnormalities of the small intestinal mucosa shown by scanning electron microscopy in infants with persistent diarrhea, although non specific, are intense enough to justify the severity of the clinical aspects displayed in a very young phase in life. Decrease in number and height of microvilli, blunting of borders of enterocytes, loss of the glycocalyx, shortening of villi and presence of a mucus pseudomembrane coating the mucosal surface were the abnormalities observed in the majority of patients. These ultra-structural derangements may be due to an association between the enteric enteropathogenic agent that triggers the diarrheic process and the onset of food intolerance responsible for perpetuation of diarrhea. An aggressive therapeutic approach based on appropriate nutritional support, especially the utilization of human milk and/or lactose-free protein hydrolyzate based formulas and the adequate correction of fecal losses, is required to allow complete recovery from the damage caused by this devastating war and for rendering peace to the small bowel environment.
